# Microsatellites reveal that genetic mixing commonly occurs between invasive fall armyworm populations in Africa

**DOI:** 10.1038/s41598-021-00298-3

**Published:** 2021-10-21

**Authors:** Amy J. Withers, Jolanda de Boer, Gilson Chipabika, Lei Zhang, Judith A. Smith, Christopher M. Jones, Kenneth Wilson

**Affiliations:** 1 Lancaster Environment Centre, Lancaster University, Lancaster, LA1 4YQ UK; 2 Rothamsted Research, Harpenden, AL5 2JQ UK; 3 University of Central Lancashire, Preston, PR1 2HE UK; 4 Zambia Agriculture Research Institute, Chilanga, Zambia; 5 Chinese Academy of Agricultural Sciences, Shenzhen, China; 6 Liverpool School of Tropical Medicine, Liverpool, L3 5QA UK; 7 Malawi-Liverpool-Wellcome Trust Clinical Research Programme, Blantyre, Malawi

**Keywords:** Ecology, Ecology, Animal migration, Invasive species, Molecular ecology, Population genetics

## Abstract

Understanding the population structure and movements of the invasive fall armyworm (FAW, *Spodoptera frugiperda*) is important as it can help mitigate crop damage, and highlight areas at risk of outbreaks or evolving insecticide resistance. Determining population structure in invasive FAW has been a challenge due to genetic mutations affecting the markers traditionally used for strain and haplotype identification; *mitochondrial cytochrome oxidase I* (COIB) and the Z-chromosome-linked *Triosephosphate isomerase* (Tpi). Here, we compare the results from COIB and Tpi markers with highly variable repeat regions (microsatellites) to improve our understanding of FAW population structure in Africa. There was very limited genetic diversity using the COIB marker, whereas using the TpiI4 marker there was greater diversity that showed very little evidence of genetic structuring between FAW populations across Africa. There was greater genetic diversity identified using microsatellites, and this revealed a largely panmictic population of FAW alongside some evidence of genetic structuring between countries. It is hypothesised here that FAW are using long-distance flight and prevailing winds to frequently move throughout Africa leading to population mixing. These approaches combined provide important evidence that genetic mixing between invasive FAW populations may be more common than previously reported.

## Introduction

The fall armyworm (FAW, *Spodoptera frugiperda*) is a highly invasive crop pest in Africa, Asia and Australasia^1^. It is native to North America where it is largely migratory, surviving winters in southern Florida and Texas before migrating north as the temperature warms, though there is some evidence that parts of Central and South America, such as Puerto Rico, have more resident populations that rarely interact with FAW from elsewhere in America^2–4^. The migratory nature of FAW means that it has a strong flight ability, and some individuals can disperse as far as 300 miles before oviposition^5^. Wherever it disperses to, the effects are devastating, causing millions of tonnes of crops to be lost, resulting in huge economic losses as well as food shortages^6^.

Understanding the migratory routes of FAW is important as these can be used to predict areas at risk and give farmers warning for early intervention techniques^2,7^. Additionally, understanding gene flow can help to predict outbreaks and foresee the spread of insecticide resistance that primarily occurs through the mixing of populations, leading to resistance alleles becoming more common in populations that were previously susceptible^7,8^.

There is currently a lot known about FAW population structure and movements in its native range (North, Central and South America), enabling farmers to deal with outbreaks and minimise crop losses^2,7,9,10^. Much less is known about potential migration and population mixing in Africa, and much of the available research has been based on *mitochondrial cytochrome oxidase I* (COIB) and the Z-chromosome-linked *Triosephosphate isomerase* (Tpi) haplotypes^9,11,12^. There are two Tpi markers used for FAW, TpiE4 that is based on variation in exon 4 which can differentiate between the corn and rice strains, and TpiI4 that is based on intronic variation and has six recorded haplotypes (five corn, one rice) that can differentiate between strains and populations^9,11,12^. However, there is some disagreement between COIB and TpiE4 haplotypes in FAW in Africa for strain identification, with evidence suggesting that the COIB haplotypes are less reliable in distinguishing between invasive populations across Africa and Asia^9,11,13^. This disagreement means that most individuals are identified as the rice strain with the COIB marker, and the same individuals are then identified as the corn strain with the TpiE4 marker. Given that the majority of samples have been collected on maize plants it has been suggested that the most accurate marker is likely to be the TpiE4 marker^9,11,13^. However, this confusion with the COIB and TpiE4 markers is most likely due to the hybridization of the corn and rice strains which has occurred in the invasive populations since FAW left its native range^14^. Furthermore, the COIB and Tpi markers show very little variation in the invasive FAW populations, for example, only two COIB haplotypes (COIB-RS, CSh4) and four TpiI4 haplotypes (TpiCa1a, TpiCa2a, TpiCa2b, TpiRa1) were previously reported in South Africa and India^12,13^.

There is also strong evidence that the invasive populations in Asia are originally from Africa, with FAW from both continents showing similar haplotype frequencies and the same mutation affecting the COIB strain identification marker^11–13^. Understanding the source of the FAW population via population genetics approaches is, therefore, an important area of research in curbing further spread, and this study addresses this by using microsatellites to determine population structure and genetic diversity across Africa.

Microsatellites are highly variable, repeat regions of DNA that are useful when studying genetic mixing within insect populations at continental scales. For example, in two hoverfly species (*Episyrphus balteatus* and *Sphaerophoria scripta*) microsatellites revealed high levels of genetic mixing, suggesting frequent migratory movements across Europe over a very large geographical scale, predominately occurring along the North–South axis^15^. Additionally, microsatellites can also be used to detect genetic differentiation at much smaller scales, such as between reduviid bugs (*Triatoma dimidiate*) in neighbouring villages in Guatemala^16^.

Microsatellites have previously been identified in FAW using populations from Texas, Mississippi, Puerto Rico and Brazil^17,18^. These microsatellites were variable enough to distinguish between three genetically distinct populations and were able to identify migrants between those populations^18^. Therefore, considering the limited variability and confusion around the COIB and Tpi haplotypes, FAW microsatellites might be a better way to identify population structure in FAW across Africa.

To improve current understanding of population movements of FAW in Africa, in this study we explored population genetic structure across FAW larvae collected from six African countries (Malawi, Rwanda, Kenya, Sudan, Kenya and Ghana) between 2017 and 2019. We used traditional strain and haplotyping methods for FAW (COIB, TpiE4 and TpiI4), as well as eight highly variable FAW microsatellite loci to determine genetic structure and mixing across countries.

## Results

### Strain identification and haplotyping using COIB and TpiE4 markers

**Figure 1:**
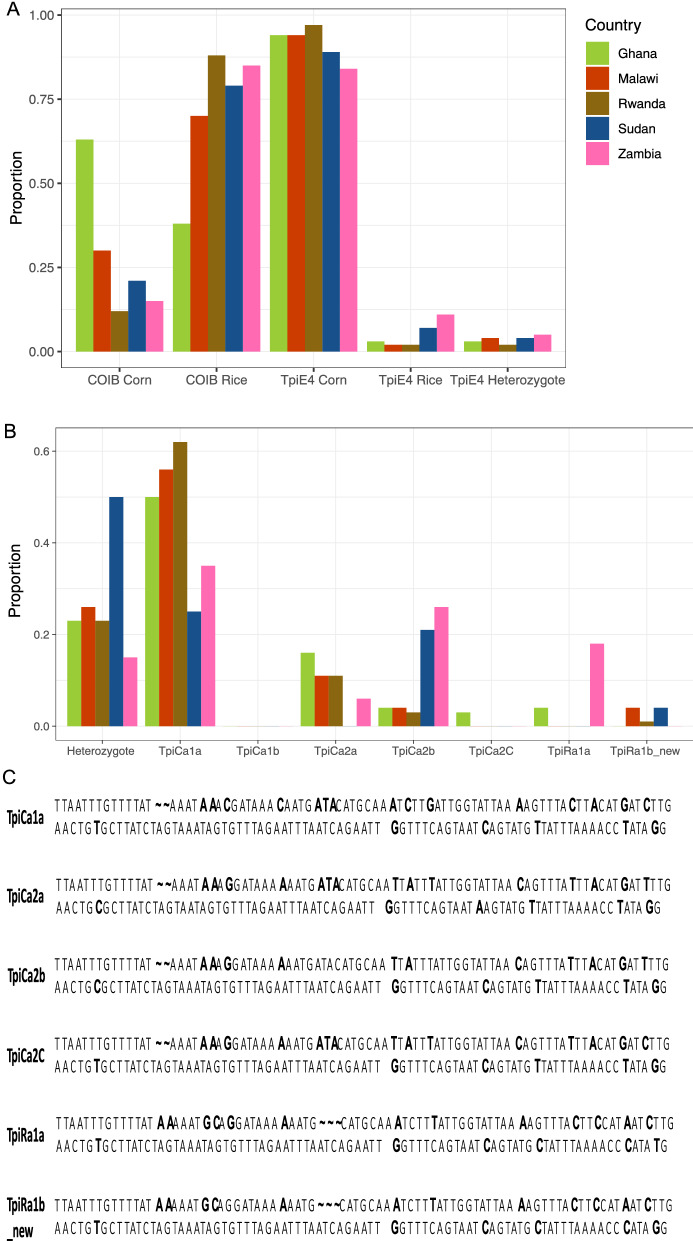
Strain and haplotype identification of FAW larvae using COIB, TpiE4 and TpiI4 markers. (**A**) Strain identification of FAW larvae using COIB and TpiE4 markers. The number of samples for COIB:TpiE4 markers tested per country are Ghana 72:72, Malawi 40:95, Rwanda 127:126, Sudan 28:28 and Zambia 53:44. (**B**) Proportion of each TpiI4 haplotype identified. (**C**) Sequences of each TpiI4 haplotype identified, variable positions are shown in bold. The number of samples for the TpiI4 marker for each country are Ghana N = 70, Malawi N = 27, Rwanda N = 141, Sudan N = 24 and Zambia N = 34.

The COIB marker was analysed using an enzyme based PCR assay^19^, and the TpiE4/TpiI4 product was sequenced using Sanger sequencing. The expected strain discordance between the COIB and TpiE4 markers was observed in all countries, with the markers only reporting the same strain in 19% of samples (see Supplementary Table S1 online). In all countries, both markers identified larvae of the corn and rice strain (Fig. 1). Overall, the COIB marker most frequently reported samples as the rice strain (mean ± S.E. = 72% ± 0.09), whereas the TpiE4 marker reported them as the corn strain (mean ± S.E. = 92% ± 0.02). Both markers showed very similar strain frequencies across Malawi, Rwanda, Sudan and Zambia. In Ghana, more samples were reported as corn strain (63%) than rice using the COIB marker compared to the other countries, and there was significant variation in the distribution of the corn strain based on the COIB marker (χ_4_ = 53.17, *P* < 0.001). However, when using the TpiE4 marker, the proportion reported as the corn strain was similar across all five countries (χ_4_ = 1.16, *P* = 0.885). Those larvae identified as corn strain by the COIB marker in Ghana (N = 45), Rwanda (N = 16), Sudan (N = 6) and Zambia (N = 8) were sequenced using Sanger sequencing to determine the haplotype. All larvae were identified as CSh4 suggesting very little genetic differentiation based on COIB haplotypes.

In all countries, the intronic TpiI4 marker identified both corn and rice strain FAW, with the corn strain (82–99%) being more common compared to the rice strain (1–18%) (Fig. 1A). The most common haplotype in every country was TpiCa1a, and the rarest was TpiCa2C (Fig. 1B). A novel rice haplotype (TpiRa1b) was identified in samples from Malawi, Rwanda and Sudan, where no larvae were of the previously recorded rice haplotype (Fig. 1B,C). The greatest number of different haplotypes was observed in Ghana, with four different haplotypes identified (TpiCa1a, TpiCa2a, TpiCa2C, TpiRa1a). Heterozygotes were recorded in all countries, however due to ambiguity in which haplotype combinations these were, they were only identified as heterozygotes (Fig. 1B). An amova was carried out on the TpiI4 alignment based on genetic distances and showed significant differences between the six countries, however the total variance explained by differences between countries was low, with most of the genetic variation being between individuals within countries, which would suggest a largely panmictic population (Table 1). To further check for genetic structuring based on TpiI4 markers, a PCA was carried out using the genetic distance between sequences and this showed clustering based on strain identification, but no evidence of structuring between the six countries (Supplementary Fig. S1A online).

**Table 1:** Results of an amova to analyse differences between the six countries based on TpiI4.

Variation	Df	Sum of Squares	Variance components	Total variance (%)	*P* value
Between countries	4	13.40	0.04	3.51	0.002
Between individuals within countries	292	342.66	1.17	96.49	NA
Total	296	356.06	1.22	100	NA

*P* value was calculated using a randomization test with 999 permutations.

### Microsatellite locus information

Microsatellites were amplified by PCR individually, and then genotyped on a ABI3500 sequencer. All eight microsatellites successfully amplified, and the number of alleles found ranged from 3 to 13 (Table 2). Twenty-one individuals (23%) had missing allele data, this ranged from 1 to 3 loci per individual, with an average of 0.34 (Table 2). Null allele frequencies were high for four alleles: Spf1502, Spf343, Spf997 and Spf670 (Table 2). Seven of the eight microsatellites significantly deviated from *Hardy–Weinberg equilibrium* (HWE) when all individuals were considered together (Table 2). However, some of these microsatellites were in HWE at the within-country level (see Supplementary Table S2 online). The *index of association* (rbarD) metric measures how likely individuals are to be the same at one particular locus in relation to other loci, and how this compares to other individuals, and can give a good indication of linkage between loci^20^. This metric was calculated and suggested a possible high chance of linkage between three pairs of loci (Spf1592 and Spf1502, Spf1592 and Spf997, Spf918 and Spf997) (see Supplementary Fig. S2 online). However, a composite linkage disequilibrium test, which measures the association between two alleles^21^, did not find any significant evidence of linkage disequilibrium (see Supplementary Table S3 online).

**Table 2:** Locus and allele information for each of the eight microsatellites, and HWE results.

Locus	Individuals	Number of alleles	Number of individuals with missing data	Allele size range (bp)	Null allele frequency	Hardy–Weinberg equilibrium *P*
Spf1502	82	10	10	124–141	*0.62*	** < 0.001**
Spf789	86	13	5	182–199	0.11	** < 0.001**
Spf343	91	8	1	107–127	*0.35*	** < 0.001**
Spf997	90	7	2	79–113	*0.23*	** < 0.001**
Spf1706	91	3	1	118–126	0.16	** < 0.001**
Spf1592	87	11	5	187–217	0.00	**0.048**
Spf918	88	6	4	111–123	0.00	0.578
Spf670	90	7	2	128–152	*0.45*	** < 0.001**

Those loci with high null allele frequencies are in italics. Loci which significantly deviate from HWE are in bold and were calculated using a Monte Carlo Exact Test.

### Population differentiation based on microsatellites

Population differentiation can be measured in several ways; here we used three common measures (Nei’s *Gst*, Hedrick’s *Gst* and *Jost’s D*) and each suggested that there was very little evidence of population differentiation across Africa^22–24^ (Table 3). In all three measures tested, a value of 0 suggests little genetic differentiation (panmixia) and 1 suggests high levels of segregation. The range of the three measures across all loci was 0.03 to 0.14 (Table 3). There was also evidence of low genetic variance based on *Fst* between countries at each locus tested (Table 3). Pairwise *Fst* values between the six countries ranged from − 0.02 to 0.08 (mean ± S. E. = 0.03 ± 0.01) suggesting high levels of population mixing (Supplementary Table S4 online). The level of inbreeding occurring within populations can be inferred from *Fis*, however, this varied between loci with high levels suggested for some loci (e.g. Spf1502 and Spf670), but low for others (e.g. Spf1592 and Spf789) (Table 3). These results suggest that in Africa, FAW may frequently mix with FAW from other countries suggesting that very little population differentiation is occurring.

**Table 3:** Genetic differentiation measures for FAW in Africa based on the eight microsatellites.

Locus	Heterozygosity		Population differentiation			F-statistics			
	*Hs*	*Ht*	*Nei’s Gst*	*Hedrick’s Gst*	*Jost’s D*	*Fst*	*Fis*	*CI Fis ( −)*	*CI Fis (* +*)*
Spf1502	0.76	0.82	0.07	0.36	0.30	0.05	0.75	0.19	0.66
Spf789	0.79	0.89	0.11	0.60	0.54	0.10	0.10	0.03	0.52
Spf343	0.74	0.75	0.01	0.05	0.04	0.00	0.52	0.19	0.61
Spf997	0.67	0.69	0.04	0.14	0.10	0.03	0.35	0.13	0.44
Spf1706	0.18	0.18	0.03	0.04	0.01	0.02	0.26	0.07	0.46
Spf1592	0.85	0.86	0.01	0.10	0.09	0.01	− 0.01	0.06	0.54
Spf918	0.64	0.65	0.01	0.03	0.02	0.01	− 0.02	0.19	0.66
Spf670	0.79	0.80	0.01	0.05	0.04	− 0.01	0.63	0.03	0.52
All	NA	NA	0.04	0.14	0.03	0.03	0.33	NA	NA

In all three measures tested, a value of 0 suggests very little genetic differentiation (panmixia) and 1 suggests high levels of segregation. All measures are based on *Hs* (heterozygosity within populations) and *Ht* (heterozygosity without population structure). F-statistics represent genetic variance in a subpopulation compared to the whole (*Fst*—values closer to 1 suggest high levels of differentiation between populations) or in a subpopulation compared to individuals within that subpopulation (*Fis*—values close to 1 suggest high levels of inbreeding in populations). Negative values of *Fst* and *Fis* should be interpreted as 0 and suggest very low differentiation of populations (*Fst*) or very low chance of inbreeding (*Fis*). Confidence intervals of *Fis* based on bootstrapping are also provided.

Population differentiation was further analysed using an amova to determine if the genetic distance between individuals varies by country, location within country or sampling year (see Supplementary Table S5 online). There was no significant difference between samples from locations within countries (F_4,81_ = 1.17, *P* = 0.120), or between sampling years (F_1,81_ = 0.96, *P* = 0.543). The amova suggested, however, that FAW from each country were genetically different to FAW from other countries (F_5,74_ = 1.86, *P* = 0.001).

As *Country* was the only significant factor influencing FAW population differentiation, a second amova was carried out to determine genetic variation between and within countries. This suggested significant differences between the six countries, however the total variance explained by differences between countries was low, and most of the genetic variation was found within individuals which would suggest a largely panmictic population (Table 4). To further check for genetic structuring based on the microsatellite markers, a PCA was carried out using the genetic distance between individuals and this showed no evidence of structuring between the six countries (Supplementary Fig. S1B online).

**Table 4:** Results of an amova to analyse differences between the six countries in this analysis based on the microsatellites.

Variation	Df	Sum of squares	Variance components	Total variance (%)	*P* value
Between countries	5	69.24	0.197	3.25	0.001
Between individuals within countries	86	672.08	1.961	32.41	0.001
Within individuals	92	358.17	3.893	64.34	0.001
Total	183	1099.49	6.051	100	NA

*P* value was calculated using a randomization test with 999 permutations.

### Population clustering based on microsatellites

**Figure 2:**
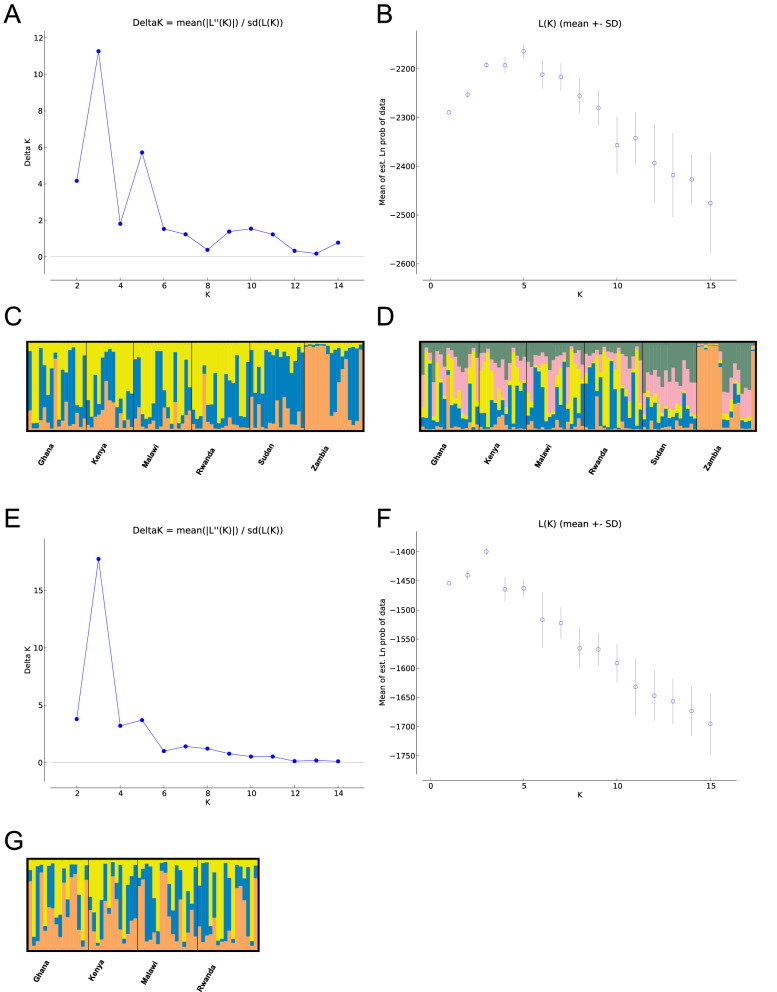
Genetic structure of FAW as assigned by *STRUCTURE* analysis of microsatellites. Panels (**A**) to (**D**) show the results of STRUCTURE with all six countries. Panel (**A**) shows the *DeltaK*, Panel (**B**) shows the *LnPr(K)* for each cluster. Panel C shows the admixture plot for the three genetic clusters based on *DeltaK*. Panel D shows the admixture plot for 5 genetic clusters based on *LnPr(K)*. Panels E to G show the results of STRUCTURE carried out to assess substructure hierarchically. Panel (**E**) and (**F**) show the *DeltaK* and *LnPr(K)* for each cluster respectively. Panel (**G**) shows the admixture plot for the three genetic clusters based on both *DeltaK* and *LnPr(K)*.

Clustering was carried out using an admixture model in *STRUCTURE*, with the number of clusters selected using *Delta K* (Evanno method) and *LnPr(K)* methods^25^. Based on *Delta K* there were three genetically distinct clusters in FAW (Fig. 2A,B). Based on *LnPr(K)*, the most likely number of clusters was five (Fig. 2C,D). In both 3 and 5 cluster scenarios, FAW from Sudan and Zambia were more genetically isolated from the four other countries, though some individuals from the other four countries do show similar assignment patterns suggesting population mixing does occur between all countries. Samples from Ghana, Kenya, Malawi and Rwanda appear very similar to each other, suggesting high levels of population mixing between these countries (Fig. 2C,D, clustering with two and four clusters is shown in Supplementary Fig. S3 online). Based on the similarities between the structuring results for 3 and 5 clusters, and that the *LnPr(K)* begins to plateau after K = 3 we propose that population structure of FAW in Africa is best described by three genetic clusters. To identify potential substructure in FAW from the four countries exhibiting evidence of genetic similarity (Ghana, Kenya, Malawi and Rwanda), a separate analysis was performed in *STRUCTURE*. This identified 3 genetic clusters as the most likely scenario, based on both *DeltaK* and *LnPr(K)*, and further confirmed high levels of mixing between the countries, with no strong evidence of substructure identified (Fig. 2E–G).

**Figure 3:**
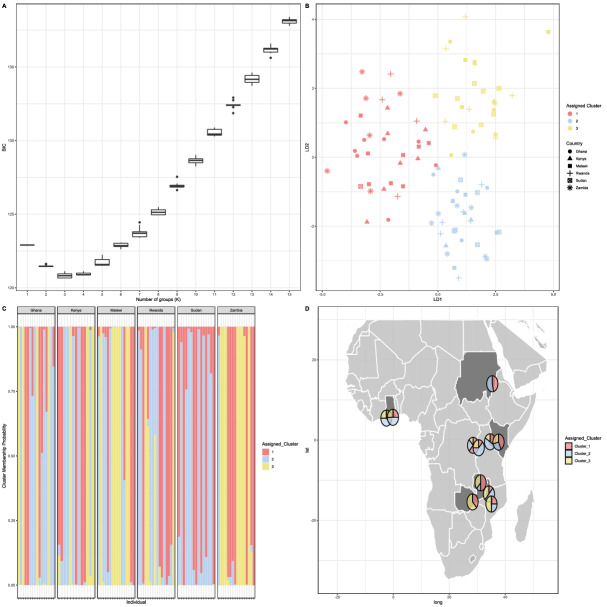
*DAPC* clustering (k = 3) and assignment of individuals from each country based on microsatellites. (**A**) The lowest BIC represents the best number of clusters, which here is 3. (**B**) The position of individuals on the first two principal components, and in (**C**) the membership probability of individuals to that cluster. (**D**) Assigned clusters for each sampling location across Africa.

*STRUCTURE* has been shown to miss some subdivision when clustering individuals^26^, therefore, population clustering analysis was also carried out by identifying clusters *de-novo* (i.e., with no prior population information provided) and then using *Discriminant Analysis Principal Components (DAPC)*. This approach determines the number of possible clusters by running successive K-means clustering, and selecting the most suitable cluster based on Bayesian Information Criterion and the number of PCs to keep was calculated to be 7 using the a-score^26^. This method identified three clusters as the best model based on BIC (BIC = 120.67) in FAW (Fig. 3A). Based on three clusters, FAW from Sudan were more genetically different to populations from elsewhere in Africa with no individuals assigned to cluster 3, whereas, cluster 3 individuals were found in all other countries (Fig. 3B–D). The three clusters highlighted similarities between the adjacent countries of Zambia and Malawi, with 50% and 44% of individuals respectively from these countries assigned to cluster 3, and similarities between Kenya and Rwanda, with 23% and 19% of individuals assigned to cluster 3 respectively (Fig. 3B–D). Ghana showed most similarities with Kenya and Rwanda, with 25% of individuals assigned to cluster 3 (Fig. 3B–D).

## Discussion

This study is the first to use microsatellites to determine FAW population mixing and genetic diversity in Africa. Considering the limited genetic diversity and unreliability of the COIB and TpiE4 haplotypes for strain identification and the potential for confusion caused by corn and rice strain hybrids^11–14,27,28^, we sought to quantify the degree of population structuring in FAW in Africa using a more robust microsatellite approach. This revealed that microsatellites had higher levels of genetic diversity compared to the COIB and TpiE4 markers, revealing that FAW in Africa is largely a panmictic population.

The previously reported discordance between the TpiE4 and COIB markers for strain identification was mirrored in this study, with very little agreement occurring between the markers. Furthermore, based on the COIB haplotypes it was not possible to determine genetic differentiation between the countries as only COIB CSh4 was found. The intronic TpiI4 marker showed more variation between the individuals, however, the vast majority of larvae were TpiCa1a, which is in line with previous studies investigating FAW in Africa and Asia^12,13,28^. Previous work based on these markers in Africa concluded that there were significant differences between some African countries with widely separated populations being genetically distinct^28^. Whilst the findings here using the TpiI4 marker do support some evidence of genetic variation between countries, it was low, suggesting more of a panmictic population of FAW across Africa based on this marker.

The low genetic variability observed with the COIB marker, and both TpiE4 and TpiI4 markers, limit the analyses that can be carried out and reduce the likelihood of genetic differentiation between countries being detected. By using highly variable microsatellites, we were able to overcome this challenge to determine genetic differentiation between FAW from different countries in Africa, as well as some similarities, suggesting the possible presence of both resident and migratory populations of FAW throughout the continent.

Most of the microsatellites in this study were out of Hardy–Weinberg equilibrium (HWE), whereas in previous population genetics studies using microsatellites in FAW from Paraguay and Brazil, no loci were found to be out of HWE^8^. However, the deviation observed in the present study is to be expected in invasive FAW populations, which have been through a tight bottleneck, given that they probably originated from a small source population in Africa, providing further evidence of a common origin for FAW which then subsequently spread across the continent^9,27^. The microsatellites also showed evidence of a genetic bottleneck and loss of diversity in the African FAW compared to populations in Texas, Mississippi, Puerto Rico, and Brazil. For example, previously reported allele sizes for locus Spf997 were in the range of 95 to 139^8,17^, whereas in this study the allele size range for the same locus was 79 to 113. The evidence of this genetic bottleneck throughout Africa offers more evidence of a single origin population instead of multiple introduction events. It is likely that if multiple incursion events had occurred then the microsatellite size ranges observed here would have matched more closely with those previously recorded.

Although FAW in Africa are likely to have undergone a population bottleneck at the time of invasion, the range of alleles for each locus identified in this study (3 to 13) was similar to that previously reported from Paraguay and Brazil (3 to 15)^8,18^. Based on this range of alleles, previous work found genetic differentiation between northern and southern FAW populations across Brazil and Paraguay, as well as gene flow across all populations sampled^8^. This indicates that despite a recent bottleneck there is still sufficient genetic diversity in microsatellite regions to enable population genetic studies of FAW in Africa.

Populations from the six countries (Kenya, Ghana, Malawi, Rwanda, Sudan and Zambia) did not show strong signs of population differentiation when using traditional measures (Nei’s *GST*, Hedrick’s *GST* and Jost’s *D*). This indicates that these populations mix frequently, and no strong genetic structure is evident. This was supported by the amova which showed that most of the genetic variance was occurring between individuals.

This lack of population differentiation between countries provides evidence consistent with FAW undergoing long distance migratory flights in Africa creating a panmixia of populations. Additionally, there was no evidence of genetic differentiation between samples from different sampling locations within the same country, confirming that populations are mixing within countries. This has significant consequences for the evolution and spread of insecticide resistance, as resistance alleles can spread rapidly throughout each country and across Africa. This is an important finding as insecticide resistance (organophosphate and pyrethroid resistance) has already been reported in FAW in China, so is highly likely to be present in Africa^14^. Considering the key role that long-distance, migratory flights played in the rapid spread of insecticide resistance both within and across continents in the invasive cotton bollworm (*Helicoverpa armigera*)^29–32^, it is important to consider the implications of frequent, long-distance flights that seem to be occurring in FAW.

The evidence of panmixia contrasts with previous results based on the COIB and Tpi markers, which analysed FAW samples from across Africa and found evidence of genetic differentiation between geographically widespread countries^9,28^. Further investigation with microsatellites using clustering approaches show that whilst the countries included in this study are similar genetically (e.g. Kenya, Rwanda, and Ghana), others are more differentiated (e.g. Sudan). We conclude from this that genetic mixing of FAW populations is occurring widely across Africa, however, there are some FAW possibly forming resident and partially segregated populations, as seen in parts of South America and the Caribbean^7^. Alternatively, the possibility that FAW have not been in Africa long enough to evolve population differentiation should also be considered.

Previous reports based on COIB and Tpi suggested a possible east–west divide between FAW populations^9^, or no clear pattern of division between populations^28^. Our study using microsatellites found that the two most genetically distinct populations are the most northerly and most southerly populations. African countries located further south (Zambia, Malawi) showed more similarities to each other compared with countries further north (Kenya, Rwanda, Ghana and Sudan) (e.g., fewer individuals were assigned to cluster 3 in the north compared to the south). This pattern of genetic separation coincides with the known migratory routes of the congeneric African armyworm (*Spodoptera exempta*) in eastern Africa, which follow the movement of the dominant winds each season, typically moving moths towards the north-west from Kenya and northern Tanzania, and a more south-westerly movement across southern Africa from Malawi^33,34^. This is also aligned with the movement of the inter-tropical convergence zone (ITCZ), with the wind direction (and hence seasonal migration) being broadly south-easterly north of the equator and north-easterly south of the equator^34^. Based on the high levels of mixing between FAW populations alongside this evidence of some genetic structuring between northern and southern populations, it is hypothesised that FAW may also follow the movement of the dominant winds if they are migratory in Africa as, like many other insects, they rely on wind to support high-altitude long-distance flights^2,5,35,36^.

This study highlights the benefits of using multiple approaches to study genetic diversity, with evidence presented for both widespread genetic mixing between populations alongside some segregation between countries. This is most likely due to a proportion of FAW adults undergoing long-distance migratory flights whilst the remaining FAW form more sedentary, resident populations. These results provide important evidence that genetic mixing between FAW populations throughout Africa may be more common than previously reported. This has important consequences for FAW management when considering factors such as the spread of insecticide resistance and crop infestations across borders.

## Methods

### Sample collection

FAW larvae for sequencing with COIB:TpiI4:TpiE4 were collected from Ghana (N = 72:70:72, 2017), Malawi (N = 95:27:40, 2018 and 2019), Rwanda (N = 127:127:141, 2017), Sudan (N = 28:28:24, 2017) and Zambia (N = 44:53:34, 2017) and stored in ethanol. FAW larvae for microsatellite analysis were collected from Ghana (N = 16, 2017, maize), Kenya (N = 13, 2019, maize), Malawi (N = 8, 2018 and N = 8, 2019, maize), Rwanda (N = 16, 2017, maize), Sudan (N = 15, 2017, maize), and Zambia (N = 16, 2017, maize) and stored in ethanol. Full collection details are provided in Supplementary Table S1 Online for COIB and Tpi markers and Supplementary Table S5 Online for microsatellite markers.

### DNA extraction

DNA was extracted from samples following the standard protocol for tissue in the Qiagen DNeasy Blood and Tissue kit. DNA was stored in buffer AE at − 20 °C. The protocol was altered slightly for extracting DNA from larvae collected in Sudan, these modifications were 200 μl ATL and an additional 200 μl 1 × SSC before incubation and the DNeasy Spin Column was centrifuged at 13,000 RPM.

### Strain identification and haplotyping using COIB and Tpi markers

DNA was amplified for strain identification using COIB (F: 5′TACACGAGCATATTTTACATC, R: 5′GCTGGTGGTAAATTTTGATATC^27^) and TpiI4/TpiE4 (F: 5′ATGATTAGGACATCGGAGC, R:5′ATGTAATCCAGTCAATGCCTA^37^, modified by de Boer). Cycling parameters for both COIB and TpiI4/TpiE4 were 94 °C 10 min, 33 cycles of 94 °C 1 min, 55 °C 1 min, 72 °C 1 min and then a final extension of 72 °C for 5 min. Following COIB amplification, the product was incubated at 37 °C for 2 h with 1 µl EcoRV restriction enzyme and 2 µl NEBuffer to determine FAW strain. EcoRV cuts the amplicon at position 1182 bp if the sample is from the rice strain resulting in two visible bands, and does not cut for the corn strain resulting in one larger band when the product is run on a gel electrophoresis (Table 3). There are five known haplotypes of the COIB marker, these are corn h1 (A_1164_A_1287_), corn h2 (A_1164_G_1287_), corn h3 (G_1164_A_1287_), corn h4 (G_1164_G_1287_) and rice (T_1164_A_1287_)^27^ and unidirectional Sanger sequencing was used to verify the COIB corn haplotypes. Sequencing reactions contained 0.75 µL BigDye® Reaction Mix, 1.70 µL 5 × BigDye® Sequencing Buffer, 0.32 µL 10 µM Forward primer, 5–20 ng template DNA and H_2_O to supplement the reaction to 10 µL. The sequencing reaction was preincubated for 1 min at 96° C followed by 25 cycles of: 10 s at 96° C; 5 s at 50° C; 4 min at 60° C. Excess incorporated dye-terminators were removed using EDTA/Ethanol precipitation before resuspending in 13 µL Hi-Di® formamide and capillary gel electrophoresis on an ABI 3500 Genetic Analyzer. Strain identification was carried out using the Tpi marker by Sangar sequencing following the same protocol as for COIB based on nucleotide variation in exon-4 (TpiE4), where the corn strain has base C_183_, the R strain has base T_183_ and hybrids (males only) have C/T_183_^27^. Tpi Intron 4 (TpiI4) was used to determine TpiI4 haplotypes based on 18 previously recorded highly variable positions^12,37^. For sequencing analysis, raw sequences were assembled and aligned using ClustalW in BioEdit^38,39^. Statistical analysis on strain and haplotype distributions for TpiE4 were carried out in R using a Poisson GLM followed by a Chi^2^ test using the amova function. Based on the TpiI4 haplotypes, an amova was carried out using the POPPR package as this gave details of the variance explained within and between samples and populations (Kamvar et al. 2014), from which a *P* value was calculated using a randomization test with 999 permutations. The genetic distance computed for the amova was also used for Principal Components Analysis (PCA) using the *prcomp* function in R.

### Microsatellite amplification

Eight highly variable microsatellites were selected for amplification based on them showing the greatest diversity in FAW in previous studies^8,17^, the microsatellite primer details are shown in Table 5. Each sample was amplified in individual 20 µl reactions composed of 2 µl EasyTaq® Buffer, 1 µl 10 µM forward primer, 1 µl 10 µM reverse primer, 0.4 µl 10 mM dNTPs, 0.1 µl EasyTaq® DNA Polymerase, 13.5 µl H_2_O and 2 µl DNA. Amplification conditions were 95 °C for 1 min, 30 cycles of 95 °C 30 s, 60 °C 30 s, 68 °C s and a final extension of 68 °C for 5 min. Once amplified, samples were stored at 20 °C until ready for genotyping.

**Table 5:** Microsatellite primer details.

Name	GenBank identification	Simple sequence repeat (SSR)	Forward primer (5′–3′)	Reverse primer (5′–3′)
Spf343	HM752609	(TG)12	[6FAM]GTCAAAGTTTTACATGGAAGCGTG	CCCATCTGTTTGTCCACAGTAAAG
Spf670	HM752637	(CAT)5	[6FAM]GGGAGAGGTTTCTAGCTTCTACGG	GAGGAGCCTTGGTTCAATAGTGC
Spf789	HM752653	(CACAC)4	[6FAM]CGACACGTTGATTGCTCACAG	AATCTTTTATCACAATTCGCAGCC
Spf918	HM752666	(TG)6	[6FAM]GCGAAATTGTTTTAATGTGGGTTG	ACGACCTATACGGACCTTGTTACG
Spf997	HM752675	(TACA)4	[6FAM]TTGATGCATGAATTTTCAAACGAG	ATCACGTTGTGGTCCAATCAATG
Spf1502	HM752731	(CA)12	[6FAM]TTTGCAATTTTAGTTACAAACGTCCTC	TATTGATAGCCTCGTGTTTGACCC
Spf1592	HM752740	(TG)10	[6FAM]GGTTCCTGTTATCACCTGCAGTA	CTATGTAGTTTATGTTAATTCGCACGAT
Spf1706	HM752751	(AC)9	[6FAM]CCACTGTACTGTGATAAACAGATGGC	ATGATCATACAAAGTGCATCCGTG

### Microsatellite genotyping

Fragment genotyping was carried out on an ABI3500 sequencer. Each reaction was composed of 11 µl HiDi Formamide, 0.4 µl Rox500 size standard and 1 µl PCR product (Spf343, Spf997 and Spf1706) or 0.5 µl PCR product (Spf1592, Spf670, Spf789, Spf918, Spf1502). Genotyping results were viewed on Thermo Fisher Connect™. The threshold for successful amplification was > 100RFU, and for heterozygotes the minor peak was > 50% of the major peak. Alleles were called based on size measurements and peaks determined to be the same allele if size measurements were within 0.5 nucleotides of each other (for example, a size of 150.2 and 150.6 were both classed as 150).

### Microsatellite analysis

Samples with fewer than 5 microsatellites amplified were removed from the analysis. Microsatellite analysis was carried out in R (v. 4.0.3)^40^. Hardy–Weinberg equilibrium was tested using the PEGAS R package^41^. The frequency of null alleles was determined using the Chakraborty et al. (1994) formula through the POPGENREPORT R package^42^. Heterozygosity and F-statistics were calculated using the HIERFSTAT package^43^. Genetic differentiation was measured using the MMOD package (G_st_ and Jost’s D)^44^. Linkage disequilibrium was calculated using an association index using the POPPR package^45^ and by composite linkage disequilbrium using GenePop (v 4.7)^46^. An Analysis of Molecular Variance (AMOVA) to determine population differentiation based on genetic distance was carried out using the adonis2 function from the VEGAN package in R for all variables^47^ and country was looked at using an amova with the POPPR package as this gave details of the variance explained within and between samples and populations^45^, from which a *P* value was calculated using a randomization test with 999 permutations. The genetic distance computed for the amova was also used for Principal Components Analysis (PCA) using the *prcomp* function in R. To identify population clusters, a Discriminant Analysis of Principle Components (DAPC) was carried out after clusters were identified de novo (i.e., no prior location information) using the *find.clusters* function in the ADEGENET package in R^26^. Optimum number of K was selected based on BIC (Fig. 3A). The number of PCs retained in the DAPC was 7, this was determined using the a-score with the *optim.a.score* function in the ADEGENET package in R (Supplementary Fig. S4 online). STRUCTURE (v. 2.3.4) was also used to identify population clusters, using an admixture model with 100,000 burnin and 100,000 reps for K1 to K15 with 15 iterations per K^48^. STRUCTURE results were visualised using STRUCTURE HARVESTER^49^, CLUMPP^50^ and DISTRUCT^51^.

## Supplementary Information

Supplementary Information.
