# Supplementary material for: Microsatellites reveal that genetic mixing commonly occurs between invasive fall armyworm populations in Africa
Source: Sci Rep. 2021 Oct 21;11:20757. doi: 10.1038/s41598-021-00298-3 (PMC8531319; doi:10.1038/s41598-021-00298-3)
Supplement: Supplementary file 1 — Supplementary Information. [file 41598_2021_298_MOESM1_ESM.pdf]

Supplementary Information for 'Microsatellites reveal that genetic mixing commonly occurs between invasive fall armyworm populations in Africa.'

Amy J. Withers, Jolanda de Boer, Gilson Chipabika, Lei Zhang, Judith A. Smith, Christopher M. Jones and Kenneth Wilson

Table S1: Strain identification using COIB and TpiE4 markers.

Country	Location	Year	Number	TpiE4 Marker				TpiI4 Marker			COIB Marker								Markers (TpiI4 and COIB) Agree (%)
				Number	corn	rice	het	Number	corn	rice	Number	corn	rice	Number haplotyped	H1	H2	H3	H4	
Ghana	Twifo Ayaase	2017	30	30	0.93	0.03	0.03	29	0.93	0.07	30	0.83	0.17	25	0	0	0	25	0
Ghana	Aviation Farm	2017	4	4	1.00	0.00	0.00	4	1.00	0.00	4	0.75	0.25	3	0	0	0	3	3
Ghana	University Cape Coast	2017	38	38	0.95	0.03	0.03	37	0.97	0.03	38	0.45	0.55	17	0	0	0	17	16
Ghana	All	2017	72	72	0.94	0.03	0.03	70	0.96	0.04	72	0.63	0.38	45	0	0	0	45	19
Malawi	Thyolo	2018	20	20	0.95	0.05	0.00	20	0.95	0.05	20	0.35	0.65	0	0	0	0	0	8
Malawi	Nchalo	2018	20	16	1.00	0.00	0.00	7	1.00	0.00	20	0.25	0.75	0	0	0	0	0	2
Malawi	Salima	2019	30	29	0.90	0.00	0.10	NA	NA	NA	0	NA	NA	NA	NA	NA	NA	NA	NA
Malawi	Lilongwe	2019	31	30	0.93	0.03	0.03	NA	NA	NA	0	NA	NA	NA	NA	NA	NA	NA	NA
Malawi	All	2018/2019	101	95	0.94	0.02	0.04	27	0.96	0.04	40	0.30	0.70	0	0	0	0	0	10
Rwanda	Ruhango	2017	9	9	1.00	0.00	0.00	8	1.00	0.00	9	0.00	1.00	0	NA	NA	NA	NA	0
Rwanda	Nyanza	2017	10	10	0.80	0.20	0.00	9	0.89	0.11	10	0.10	0.90	2	0	0	0	2	3
Rwanda	Gisagara	2017	10	10	1.00	0.00	0.00	10	1.00	0.00	10	0.20	0.80	2	0	0	0	2	2
Rwanda	Muhanga	2017	8	8	1.00	0.00	0.00	8	1.00	0.00	8	0.13	0.88	1	0	0	0	1	1
Rwanda	Kirehe	2017	10	10	1.00	0.00	0.00	8	1.00	0.00	10	0.30	0.70	3	0	0	0	3	3
Rwanda	Gatsibo	2017	10	10	1.00	0.00	0.00	7	1.00	0.00	10	0.10	0.90	1	0	0	0	1	1
Rwanda	Kayonza	2017	10	10	0.90	0.00	0.10	8	1.00	0.00	10	0.10	0.90	1	0	0	0	1	1
Rwanda	Nyagatare	2017	10	10	0.90	0.00	0.10	10	0.90	0.10	10	0.20	0.80	2	0	0	0	2	2
Rwanda	Rusizi	2017	10	10	1.00	0.00	0.00	10	1.00	0.00	10	0.30	0.70	3	0	0	0	3	3
Rwanda	Nyamasheke	2017	10	10	1.00	0.00	0.00	10	1.00	0.00	10	0.10	0.90	1	0	0	0	1	1
Rwanda	Karongi	2017	10	9	1.00	0.00	0.00	9	1.00	0.00	10	0.00	1.00	0	0	0	0	0	0
Rwanda	Ngororero	2017	10	10	1.00	0.00	0.00	10	1.00	0.00	10	0.00	1.00	0	0	0	0	0	0
Rwanda	Kamonyi	2017	10	10	1.00	0.00	0.00	10	1.00	0.00	10	0.00	1.00	0	0	0	0	0	0

Rwanda	All	2017	127	126	0.97	0.02	0.02	141	0.99	0.01	127	0.12	0.88	16	0	0	0	16	17
Sudan	Al Qadarif	2017	28	28	0.89	0.07	0.04	24	0.96	0.04	28	0.21	0.79	6	0	0	0	6	7
Zambia	Lusaka	2017	15	15	0.73	0.20	0.07	13	0.69	0.31	15	0.47	0.53	7	0	0	0	7	6
Zambia	Western Province	2017	3	1	1.00	0.00	0.00	NA	NA	NA	3	0.00	1.00	0	NA	NA	NA	NA	0
Zambia	Eastern Province	2017	4	4	0.50	0.50	0.00	4	0.50	0.50	4	0.00	1.00	0	NA	NA	NA	NA	2
Zambia	Southern Province	2017	3	3	0.67	0.00	0.33	2	1.00	0.00	3	0.33	0.67	1	0	0	0	1	1
Zambia	Northern Province	2017	10	10	1.00	0.00	0.00	9	1.00	0.00	10	0.00	1.00	0	NA	NA	NA	NA	0
Zambia	North-West Province	2017	8	4	1.00	0.00	0.00	NA	NA	NA	8	0.00	1.00	0	NA	NA	NA	NA	0
Zambia	Luapula	2017	10	7	1.00	0.00	0.00	6	1.00	0.00	10	0.00	1.00	0	NA	NA	NA	NA	0
Zambia	All	2017	53	44	0.84	0.11	0.05	34	0.82	0.18	53	0.15	0.85	8	0	0	0	8	9

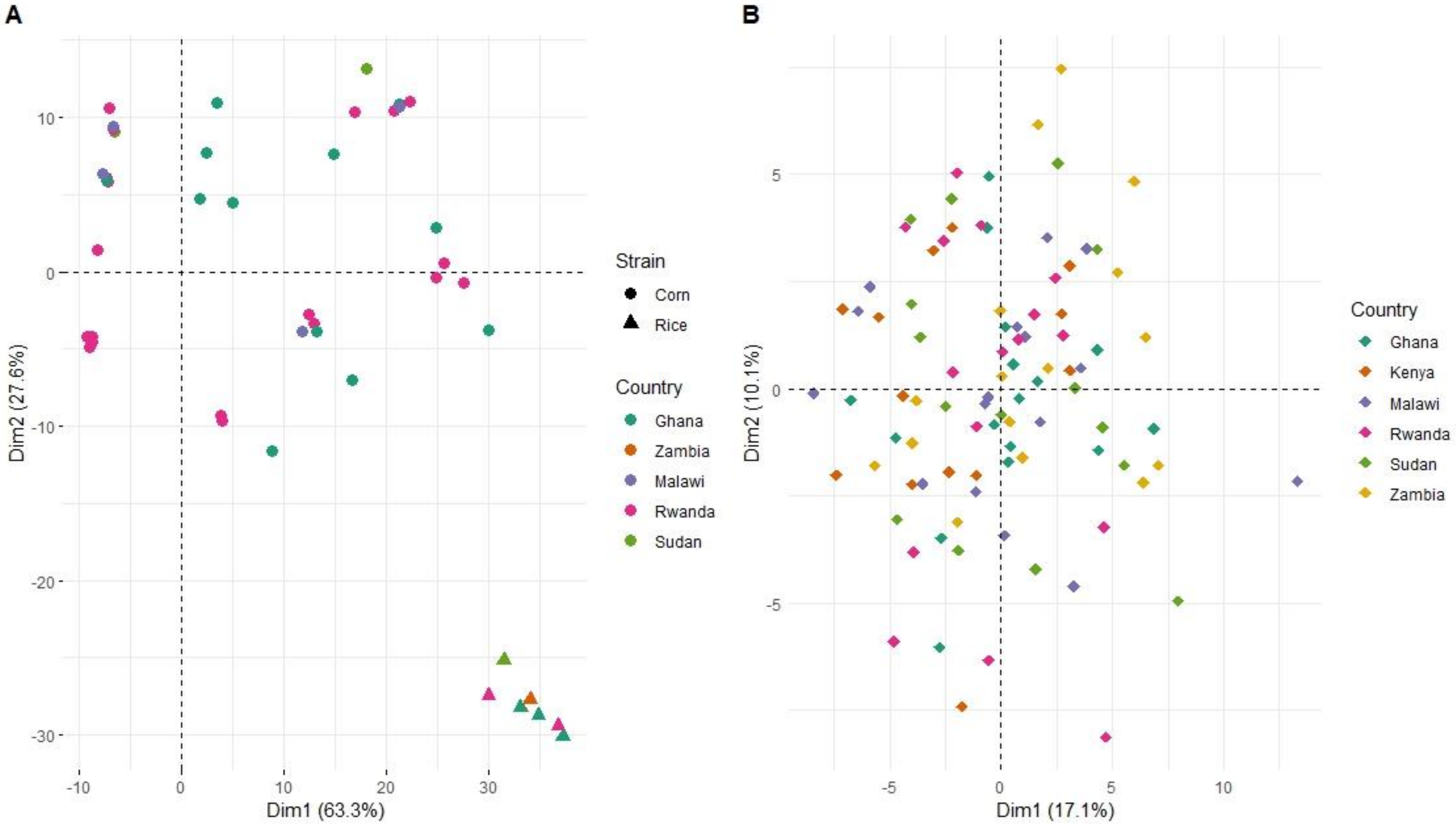

Figure S1: Principal Components Analysis on the genetic distance between individuals based on A) Tpil4 marker and B) microsatellites.

Table S2: Hardy-Weinberg equilibrium (HWE) for each locus separated by country. Locus which significantly deviate from HWE are bold and shaded grey, *P* value was calculated using a Monte Carlo Exact Test.

**Locus**	**Ghana**	**Kenya**	**Malawi**	**Rwanda**	**Sudan**	**Zambia**
	P	*P*	*P*	*P*	*P*	*P*
**Spf1502**	**<0.001**	**<0.001**	**<0.001**	**<0.001**	**0.043**	**<0.001**
**Spf789**	0.114	0.31	**0.049**	0.47	0.958	0.08
**Spf343**	**<0.001**	0.155	**0.004**	**0.002**	**0.004**	0.01
**Spf997**	**<0.001**	0.27	0.077	0.611	0.089	**<0.001**
**Spf1706**	1	1	0.32	0.071	**0.033**	1
**Spf1592**	0.705	0.716	0.264	0.122	0.727	**0.031**
**Spf918**	0.332	0.645	0.427	0.603	0.746	0.129
**Spf670**	**<0.001**	**0.001**	**0.002**	**<0.001**	**<0.001**	**<0.001**

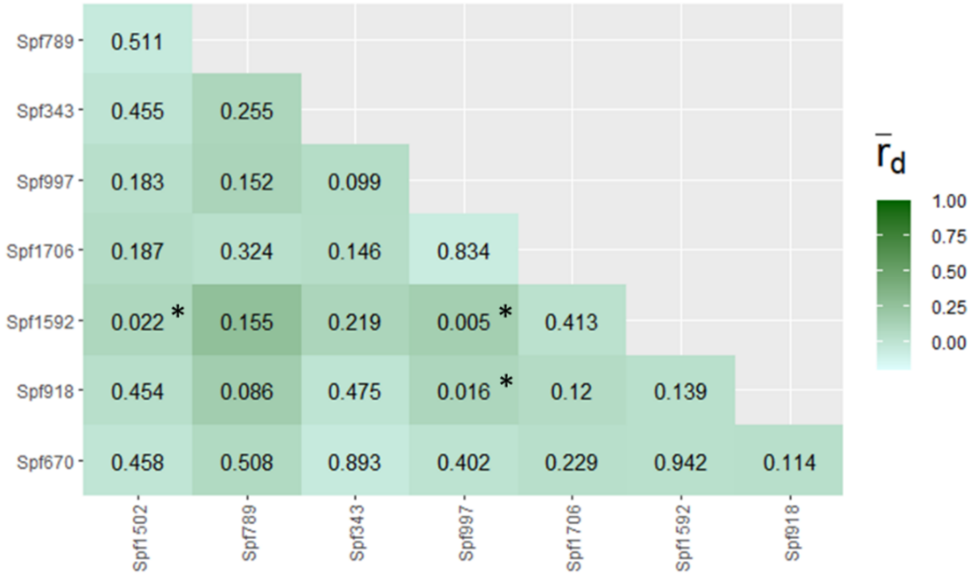

Figure S2: Standardised index of association (rbarD) for each pair of loci, with darker green representing higher index of associations. Numbers are *P* values, and those with asterisks are significant at *P*<0.05.

Table S3: Composite linkage disequilibrium *P* value for each pair of loci. No pairs showed significant evidence of linkage between alleles.

	**Spf1502**	**Spf789**	**Spf343**	**Spf997**	**Spf1706**	**Spf1592**	**Spf918**	**Spf670**
**Spf1502**								
**Spf789**	0.921							
**Spf343**	0.998	0.942						
**Spf997**	0.196	0.797	0.547					
**Spf1706**	0.568	0.700	0.828	0.968				
**Spf1592**	0.982	1.000	0.076	0.999	0.568			
**Spf918**	0.707	0.268	0.058	0.726	0.565	0.414		
**Spf670**	0.088	0.849	0.065	0.403	0.949	0.778	0.788	

Table S4: Pairwise *Fst* values for the six countries. Pairwise *Fst* values were calculated based on Nei's method (Nei, 1987).

	**Ghana**	**Kenya**	**Malawi**	**Rwanda**	**Sudan**
**Kenya**	**-0.023**				
**Malawi**	**-0.004**	**-0.001**			
**Rwanda**	**0.001**	**-0.014**	**0.008**		
**Sudan**	**0.043**	**0.041**	**0.055**	**0.045**	
**Zambia**	**0.070**	**0.052**	**0.071**	**0.082**	**0.079**

Table S5: Results of an amova to determine if the genetic distance of FAW in Africa is influenced by the country they are from, the location they were sampled from within country or the year they were sampled.

**Variable**	**Df**	**Sum of Squares**	**R^2**	**F**	***P***
Country	5	136.08	0.01	1.86	**0.001**
Year	1	14.04	0.01	0.96	0.543
Location	4	68.46	0.488	1.17	0.120
Residual	81	1184.16	0.84		
Total	91	1402.74	1		

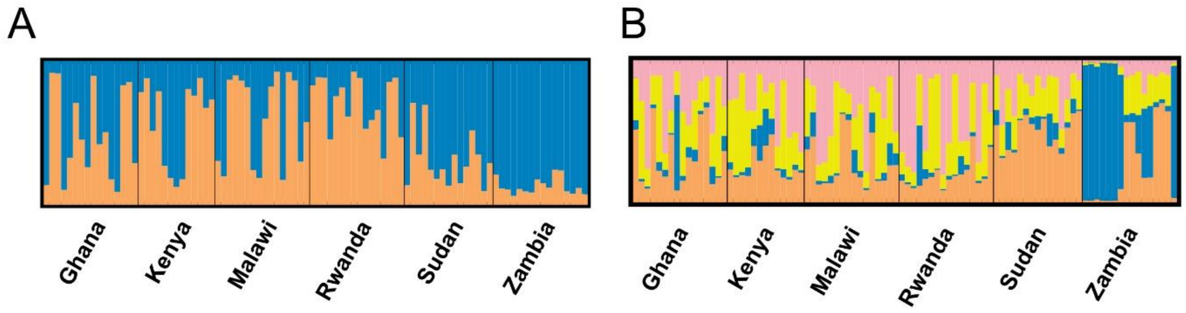

Figure S3: Genetic structure of FAW as assigned by *STRUCTURE* analysis of microsatellites. Panel A shows the assignment of individuals based on 2 genetic clusters, and panel B shows the assignment of individuals from each country based on 4 genetic clusters.

Table S5: FAW larvae collection details for microsatellite analysis. All larvae were collected at instar 3 or 4. If the coordinates were not recorded, then it was estimated using a central point for the nearest named town or province. Microsatellites were amplified from 16 samples in each country. N shows the number of samples included in the study.

Country	Location	Collector*	N	Crop	Date	Storage	Longitude	Latitude
Ghana	UCC	A	8	Maize	16/10/2017	Ethanol at -20°C	-0.16	5.68
Ghana	Twifo Ayaase	A	8	Maize	14/08/2017	Ethanol at -20°C	-1.49	5.47
Kenya	Embu	B	7	Maize	19/06/2019	Ethanol at -20°C	34.58	-0.43
Kenya	Homa Bay	B	6	Maize	19/06/2019	Ethanol at -20°C	37.60	-0.49
Malawi	Salima	C	8	Maize	28/01/2019	Ethanol at -20°C	34.15	-13.4
Malawi	Thyolo	C	8	Maize	17/09/2018	Ethanol at -20°C	35.07	-15.92
Rwanda	Kayonza	D	8	Maize	03/05/2017	Ethanol at -20°C	30.51	-1.91
Rwanda	Nygatare	D	8	Maize	05/05/2017	Ethanol at -20°C	30.33	-1.29
Sudan	Al Qadarif	F	15	Sorghum	01/09/2017	Ethanol at -20°C	35.38	14.04
Zambia	Northern Province	E	8	Maize	15/05/2017	Ethanol at -20°C	31.19	-10.65
Zambia	Lusaka (near Lufansa)	E	8	Maize	20/01/2017	Ethanol at -20°C	28.32	-15.4

*A: Ben Mensah, B: Aislinn Pearson, Kentosse Ouma, Catherine Adongo Awuoche and Sevgan Subramanian, C: Donald Kachigamba and Amy Withers, D: Patrick Karangwa, and Bellancile Uzayisenga, E: Gilson Chipabika, Miyanda Moonga, Phillip Nkunika and Kenneth Wilson F: Guillaume Sneessens

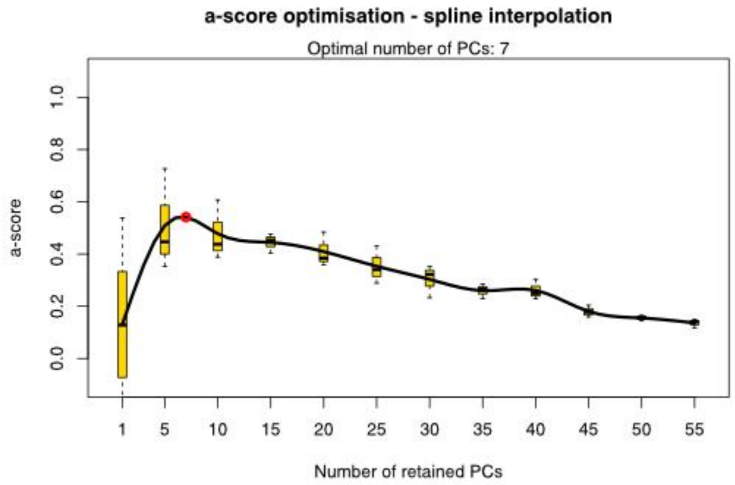

Figure S4: The results of the a-score optimisation test to determine the number of principal components to retain in the DAPC analysis.
